# PRICE: a personalized recursive intelligent cost effectiveness analysis framework for rare disease diagnosis

**DOI:** 10.1186/s12911-025-03277-0

**Published:** 2025-11-26

**Authors:** Mengshu Nie, Yujing Yao, Junyoung Kim, Cong Liu

**Affiliations:** 1 Division of Genetics and Genomics, Department of Pediatrics, Boston Children’s Hospital, Boston, MA USA; 2 Gertrude H. Sergievsky Center, Columbia University Irving Medical Center, New York, NY USA; 3 Children’s Hospital Informatics Program, Boston, MA USA

**Keywords:** Diagnostic workflow modeling, Cost-effectiveness analysis, AI-delegated diagnostic procedure

## Abstract

**Background:**

Rare disease diagnosis often involves complex, lengthy, and costly procedures. Traditional cost-effectiveness analyses typically rely on static diagnostic workflow models that apply uniform diagnostic strategies across heterogeneous patient populations. With recent advancements in artificial intelligence (AI) and a growing emphasis on personalized medicine, there is a pressing need for dynamic frameworks that assess diagnostic cost-effectiveness at the individual patient level.

**Methods:**

We introduce the PRICE analysis framework, a novel, tree-based model designed to evaluate the cost-effectiveness of diagnostic strategies, accommodating both expert-alone and AI-delegated decision-making modes. The model computes the expected cost of a diagnostic process via a back-propagation algorithm and quantifies effectiveness through a utility-based approach (i.e., *Quality Adjusted Life Years*). Parameters such as disease prevalence, test costs, test performance metrics, and turnaround time are incorporated to enable individualized assessments.

**Results:**

We demonstrat the utility of this novel framework in a proof-of-concept study by evaluating four diagnostic strategies for developmental delay (DD) and multiple congenital anomalies (MCA). The results highlight how PRICE can support personalized decision-making by modeling outcomes under varying parameters such as cost, prevalence, yield, and AI accuracy. To better visualize and interpret this framework, we developed an interactive web-based tool to demonstrate how to build PRICE pathways and conduct cost-effectiveness analysis in real time.

**Conclusion:**

PRICE is a novel cost-effective analysis framework that captures the sequential and recursive nature of real-world diagnostic workflows, with the ability to be extended to future AI-integrated clinical practice. It enables personalized evaluations of diagnostic strategies from both economic and clinical perspectives, promoting more informed and individualized decision-making for rare disease diagnosis.

## Background

The modern diagnostic process is an iterative cycle of information gathering, interpretation and the selection of diagnostic strategies. For individuals with rare genetic disorders, diagnostic workflows often follow a multitiered approach in which each tier progressively refines the diagnostic hypothesis [1]. The process usually starts with an initial screening to gather preliminary clinical information, followed by more targeted investigations that provide deeper insights into the patient’s condition. Additional tiers of testing may be conducted to reconfirm a suspected diagnosis or to explore alternative possibilities if the previous tests are inconclusive[2]. Determining which tests to perform and in what sequence is therefore a critical aspect of diagnostic decision-making, directly affecting both the accuracy and efficiency of reaching a diagnosis [3, 4]. Schaafsma et al. [5] outlined four key dimensions to evaluate diagnostic tests: test characteristics, added value of the test, clinical outcomes and cost-effectiveness. Newman-Toker et al. [6] also emphasized the importance of economic evaluation in optimizing diagnostic quality, particularly in preventing the overuse or underuse of tests.

The existing literature on clinical cost-effectiveness reveals several limitations. First, mostevaluations tend to focus on isolated tests or interventions, with limited attention to the multi-tiered diagnostic pathways commonly employed for rare diseases. As most of these studies are designed around specific diagnostic procedures, there remains a critical need for generalized frameworks that can be systematically applied across diverse diagnostic strategies. Second, current guidelines for cost-effectiveness analysis are often framed from the healthcare provider or societal perspective, overlooking patient-specific circumstances and preferences, which leads to generalized analyses that may not align with individual patient welfare [7]. For example, Li et al. [8] evaluated the cost-effectiveness of multiple genetic testing strategies for developmental delays (DD) and multiple congenital anomalies (MCA) , comparing genome-wide and exome sequencing (GS and ES) with standard chromosomal microarray analysis (CMA) and gene panel tests. This analysis implicitly assumes one-size-fits-all strategies across all patients within a clinical setting. With a growing focus on personalized medicine, especially with the rise of artificial intelligence (AI) to tailor diagnostic and treatment plans for individuals [9], there is an increasing demand for diagnostic strategies that adapt to individual patient characteristics [10]. This shift highlights the need for individual level cost-effectiveness analysis frameworks, guiding test selection based not on population averages but rather on the expected value for a specific patient.

Achieving such a personalized diagnosis requires information from multiple sources, such as the pretest probability of disease, the yielding rate of a given test, the patient’s phenotypes, demographics and clinical history, often within a probabilistic reasoning process [11]. Advances in AI can rapidly analyze large-scale patient data, predict diagnostic yield based on individual patient profiles, and facilitate efficient diagnostic decision-making [12–16]. The manual process of collecting these data points and conducting probabilistic reasoning can be cognitively burdensome and error-prone, which underscores the need for intelligent systems that assist or automate diagnostic reasoning [17]. One notable example of such systems is IDx-DR, an autonomous AI-based diagnostic tool for detecting diabetic retinopathy from retinal images [18]. While this system has demonstrated the promise to expand patient access to screening and accelerate referrals for high-risk patients, challenges such as obtaining high-quality images [19] and the need to redesign clinical pipeline logistics [20] still remain. Further, the human-AI collaboration models have shown substantial potential in augmenting healthcare professionals’ cognitive strength by leveraging AI’s analytical capabilities, while preserving expert oversight and final judgment [21, 22]. Building on the importance of patient-centered diagnostic testing and the growing potential of human–AI collaboration, we introduce a novel tree-based cost-effectiveness analysis framework and present a proof-of-concept case study demonstrating its applicability across diagnostic processes, including scenarios where AI assists in decision-making.

## Methodology

### Conceptualized framework

**Fig. 1:**
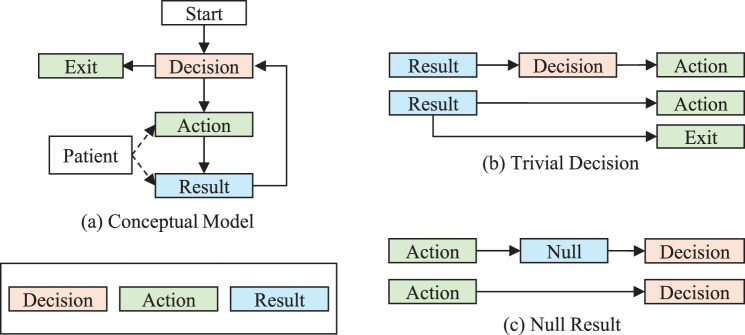
The diagnostic process. a) A general diagnostic process follows an iterative decision-action-result sequence. For example, a patient with developmental delay needs to decide between two genetic tests (decision node), gene panel or exome sequencing (action nodes). After taking the test and obtaining the test result (result node), the patient may need to decide for further actions or exit the process (exit node) if no more action is needed. Note that the exit node can be considered a special type of action; however, we distinguish it explicitly to indicate that it represents the termination point of the diagnostic process. b) A decision node can be neglected if there is only one action node following (e.g., only one test to be considered) or when the patient exits the process. c) A new decision node can directly follow an action node if the action does not yield any diagnostic information

We define a tree $T = \left( {N,E} \right)$ representing a diagnostic process, with $N = \{ {n_i}|i = 0,1,2, \ldots ,m - 1\}$ denoting the set of *m* nodes (root node ${n_0}$) and $E = \{ {e_{ij}}|{n_j} \in children\left( {{n_i}} \right)\}$ denoting the set of directed edges connecting each parent node to its child nodes. The set of leaf nodes is defined as $\{ {n_l} \in N | children\left( {{n_l}} \right) = \emptyset \}$. Figure 1a illustrates the generalized conceptual model of a diagnostic process. To capture the logic and structure of a real-world diagnostic workflow, we define three types of nodes in this tree, *decision*, *action* and *result*; each of which carries attributes including cost and turnaround time. A decision node (*D*) acts as a clinical judgment or planning point at which the next clinical action will be selected among multiple options based on available information, such as the patient’s phenotypes or the prior test results. The decision node is said to be *trivial* when followed by a single action node (Fig. 1b). An action node (*A*) corresponds to the execution of a diagnostic step, such as taking a specific test, attending a genetic consultation, or simply exiting the process. It may transition directly to a new decision node if it does not yield any diagnostic information (e.g., a genetic consultation; Fig. 1c). A result node (*Y*) takes either positive (*1*) or negative (*0*) values for a dichotomous diagnostic test or continuous numeric values for a quantitative test. These nodes collectively define the sequential and recursive nature of a diagnostic pathway. The process begins with the patient entering an initial decision node, which leads to one or more action nodes, such as the selection of a diagnostic test. Upon the completion of the action, the subsequent result node directs to another decision node, evaluating whether to terminate the diagnostic procedure or proceed with the next action. The patient may exit the process if a conclusive diagnosis is reached at any stage, or if all potential actions have been exhausted; otherwise, the pathway continues recursively through additional *decision‒action‒result* cycles until an *exit* node is reached.

A fundamental property in defining this graph is the transition probability $${P_{ij}}$$ from a parent node $${n_i}$$ to its child node $${n_j}$$ which must collectively sum to one (i.e., $$\sum\nolimits_{{n_j} \in children\left( {{n_i}} \right)} {{P_{ij}} = 1} $$). A transition probability is said to be trivial if a parent node is followed by a single child node (i.e.,$$ {P_{ij}} = 1$$); thus, the transition from results to decisions is always trivial by definition. Nontrivial transition probabilities are therefore either (1) from decision to action or (2) from action to result. The former represents the likelihood of selecting a particular diagnostic actionwhich is usually dichotomized (i.e., taking values of either 0 or 1) in clinical settings where standard protocols are followed [23].However, in practice, clinicians may deviate from standardized pathways [24], allowing these probabilities to be estimated empirically from historical test selection frequencies. Transition probabilities from action to result typically correspond to test performance and disease prevalence metrics, such as the diagnostic yield of a test, estimated as the percentage of positive results out of *all individuals* tested [25]. These metrics can be obtained from clinical studies or institutional data sources. It is important to note that these probabilities are generally population-based and rarely account for individual patient variability, whereas in AI-assisted approaches they can be estimated by conditioning on patient-specific information (see Section “Extending the Framework to the AI-delegation Mode”).

### Expected cost computation

To calculate the accumulated expected cost $E(C_i^+)$ at each node ${n_i}$ , we initialize each node with a base value ${C_i}$which is the direct cost associated with the node. Decision nodes capture costs related to the expert time required todetermine the subsequent step; in practice, such decision-making costs are often related to clinical office visits. Action nodes are associated with the costs of diagnostic tests or clinical consultations, whereas result nodes, which indicate diagnostic outcomes, usually carry no cost. The cumulative expected cost at node ${n_i}$ can therefore be recursively defined as:

1 $$E\left( {C_i^ + } \right) = \left\{ {\matrix{ {{C_i}, if\, children\left( {{n_i}} \right) = \emptyset } \cr {{C_i} + \mathop \sum \limits_{{n_j} \in children\left( {{n_i}} \right)}^{} {P_{ij}}*E\left( {C_j^ + } \right), otherwise. } \cr } } \right.$$

    

The primary objective is to compute the cumulative cost at the root node, which reflects the expected total cost of the entire diagnostic process across different potential pathways.

Building upon the tree structure defined in the previous section, a recursive algorithm is developed below to compute the expected cost of a diagnostic procedure, which is conceptually analogous to the backpropagation calculation [26]. Specifically, the computation begins at the leaf nodes which is analogous to the output layer in a neural network, and proceeds recursively upward through the tree to the root node, similar to propagating gradients backward from the output to the input layers. At each step, the expected cost at a given node is updated according to the base costs and accumulated expected costs of its child nodes. This recursive nature ensures that diagnostic decisions and outcomes at the deeper layer of the tree are appropriately reflected in the cost estimation at earlier stages of the diagnostic process. The time complexity of this algorithm is O(N), where N is the number of nodes in a given tree.


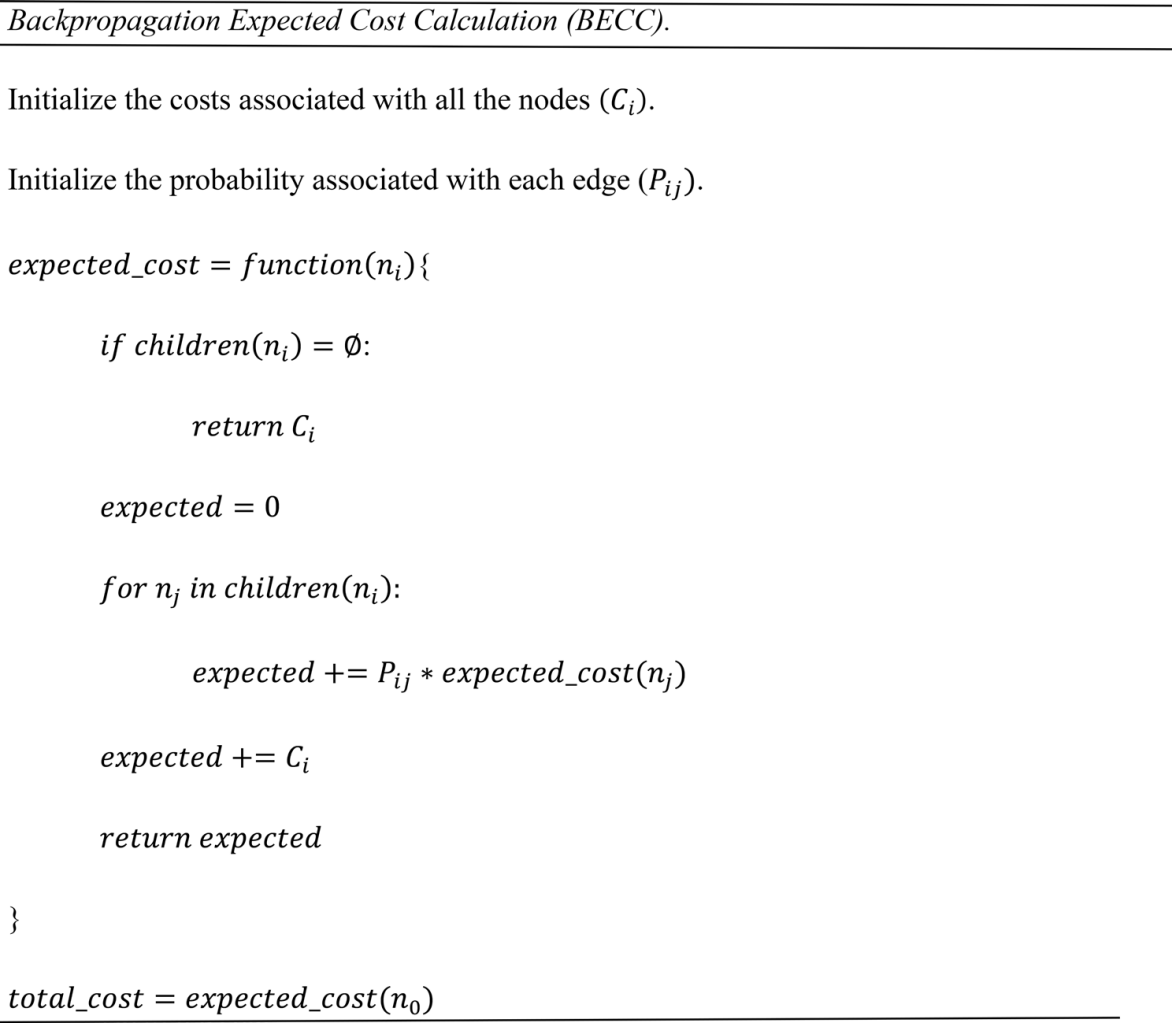


### Cost effectiveness analysis

To complement the cost-estimation algorithm detailed in the last section, we extend this framework with Quality-Adjusted Life Years (QALYs) which constitutes a complete cost-effectiveness analysis (CEA) framework. QALY captures both the quantity and quality of life by weighting time lived in various health states using utility values ranging from 0 (equivalent to death) to 1 (perfect health); values below 0 may be used to reflect states considered worse than death [27]. This utility value is typically determined through survey studies or jointly chosen by patients and physicians [28]. To allow for a nuanced evaluation of both economic burden and clinical effectiveness, we define *cost per QALY* to compare across different diagnostic strategies and facilitate more informed and personalized healthcare decision-making.

We first introduce the concept of an *outcome state* that be defined by two components: (a) the final result obtained at the the end of the diagnostic process and (b) the cumulative turnaround time $TAT$ (measured in years) required to reach thefinal diagnostic result. Formally, the outcome state at a leaf node *i* (i.e., the *exit* node of the pathway ) is expressed as ${S_i} = \left( {{O_i} ,TA{T_i}} \right)$, where ${O_i}$ indicates the outcome of the final diagnostic result node prior to exit and $TA{T_i} = \sum\nolimits_{j \in ancestors\left( i \right)} {{T_j}}$ indicates the total turnaround time accumulated across individual nodes $j$ along the pathway to the exit. Let ${S_0}$ denote the initial undiagnosed state, with utility values $U\left( {{S_0}} \right), U\left( {{S_i}} \right)$ associated with the initial undiagnosed state and outcome state. The QALY(*x*) for a patient in outcome state ${S_i}$ over the next *x* years following diagnostic initiation is formulated as

$$QAL{Y_i}( x ) = TA{T_i}*U( {{S_0}} ) + ( {x - TA{T_i}} ) *E[ {U( {{S_i}} )} ] $$

When multiple outcomes are possible for a diagnostic strategy, the overall expected QALY is computed as the probability-weighted average of QALYs across all potential outcome states.

### Extending the framework to the AI-delegation mode

**Fig. 2:**
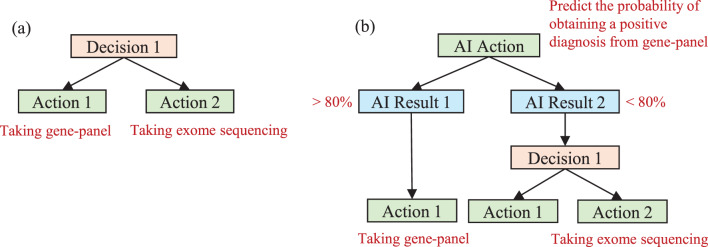
AI-delegation mode structure. a) Expert-alone decision making. The physician directly makes a choice between two actions (e.g., gene panel vs. exome sequencing). b) AI-delegated decision making. Suppose that the AI action node at the top predicts how likely it is for a gene panel test to produce a positive diagnosis. If this probability is at least 80% (AI Result 1), then the gene panel test can be directly ordered for the patient (Action 1); otherwise, a physician intervenes to decide between gene panel (Action 1) and exome sequencing (Action 2)

In traditional expert-only diagnostic workflows, achieving personalized diagnosis is inherently challenging. This is primarily because transition probabilities are rarely patient-specific and rely instead on generalized clinical guidelines. Even when clinicians tailor decisions based on an individual’s profile, it is difficult to quantify those probabilities without observing the process in action. In contrast, AI-based approaches can, by design, estimate these conditional probabilities in advance through probabilistic modeling. The *AI-delegation* mode was initially proposed by Gustavo et al. [29], essentially referring to a collaborative model where AI assists human experts in the clinical decision-making process. In the expert-alone mode, a decision node typically functions as a singular point where an expert selects the appropriate next action, as illustrated in Fig. 2a. In contrast, the AI-delegation mode introduces a more intricate node structure, as depicted in Fig. 2b. Specifically, the “decision node + *N* action nodes” configuration is now replaced by a sequence that begins with an AI action node, in which the AI predicts the optimal diagnostic test according to a specific patient’s phenotypes, demographics, and prior diagnostic outcomes such that the diagnostic procedure is customized to each individual patient. One or more AI result nodes subsequently follow the AI action nodes, which are partitioned by predetermined threshold values. Depending on the AI’s prediction, the process then proceeds to either an expert decision node, which mirrors the structure of the expert-alone mode, or a trivial decision node, which directly transitions to an action node without further expert input. The decision cost is thus omitted in the branch where an action node directly follows the AI result node, since test ordering is automated with no human intervention needed. The transition probabilities in AI-delegation mode therefore rely on the AI prediction and performance (i.e., precision; see the next case study section for an example). This expanded structure allows for more efficient AI-assisted test selection while maintaining human oversight and intervention.

## Case study: application of DPCA to the diagnosis process of DD & MCA

In this section, we apply the framework proposed in the methodology section to the diagnostic procedure of *developmental delays* (*DD*) and *multiple congenital anomalies* (*MCA*). This case study primarily serves as a proof-of-concept to demonstrate how the PRICE framework can be applied to measure cost-effectiveness of different diagnostic strategies.

### Tree representation

In accordance with the diagnostic strategies outlined by Li et al. [8], seven approaches are commonly adopted for diagnosing DD & MCA:

1. Standard testing: first-tier chromosomal microarray analysis (CMA) + second-tier targeted single-gene/gene-panel (GP).
2. Third-tier whole exome sequencing (ES) after standard testing above fails to provide a diagnosis.
3. First-tier CMA, second-tier ES if CMA fails to provide a diagnosis.
4. First-tier ES, second-tier CMA if ES fails to provide a diagnosis.
5. ES and CMA concurrently perform first-tier testing.
6. Third-tier genome sequencing (GS) after standard testing fails to provide a diagnosis.
7. GS alone is used for first-tier testing only.

For this case study, we simplify these seven strategies into the following four representative ones:

1. First-tier CMA + second-tier GP;
2. First-tier CMA + second-tier GP + third-tier ES;
3. First-tier CMA + second-tier ES;
4. First-tier ES only.

**Fig. 3:**
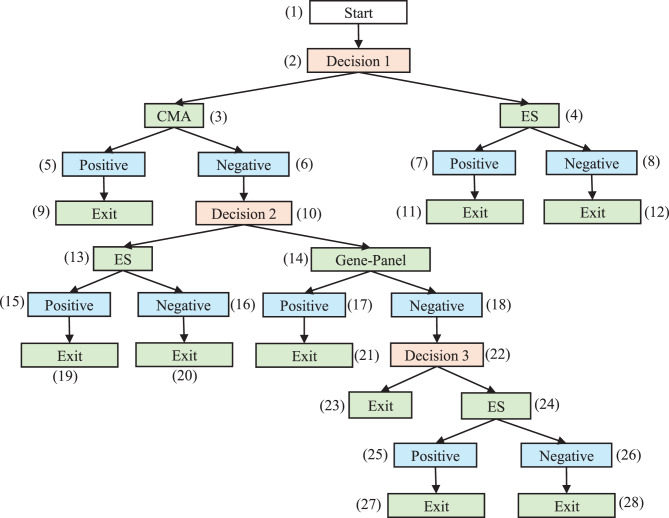
Expert-alone mode. The 4 scenarios are indicated with the following node sequences. Scenario 1 (CMA + GP): (1) – (2) – (3) – (6) – (10) – (14) – (18) – (22) – (23). Scenario 2 (CMA + GP + ES): (1) – (2) – (3) – (6) – (10) – (14) – (18) – (22) – (24). Scenario 3 (CMA + ES): (1) – (2) – (3) – (6) – (10) – (13).Scenario 4 (ES only): (1) – (2) – (4)

**Fig. 4:**
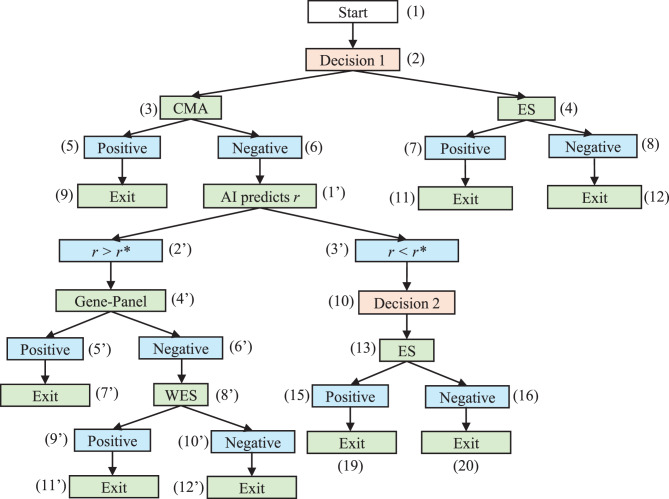
AI-delegation mode. Following the *negative* result node (6) of cma, the *decision 2* node (10) from the expert-alone mode is now replaced by the AI action node (1’) and two AI result nodes (2’ and 3’). The r > r* branch is fully AI-automated with no extra decision nodes involved. The r < r* branch returns the original *decision 2* node and is assumed to be independent of AI performance. For simplicity, we assume that only es is considered if r < r*

Figure 3 depicts the full diagnostic graph according to the four strategies listed above. Figure 4 presents a simplified workflow of the AI-delegation mode, in which the AI system assists in determining strategies (2) and (3) dynamically, i.e., whether a patient should first proceed to the GP or ES following a negative CMA result. The AI action node (1’) predicts the probability that the GP will yield a positive result. Based on a predetermined threshold value ${r^*}$, the model follows one of two paths: if $r \ge {r^*}$, the subtree under the AI result node (2’) is automatically activated, and the patient first undergoes GP before proceeding with ES (strategy 2); otherwise, the decision is to proceed with ES testing directly (strategy 3). The rationale behind this design is to utilize AI to examine whether the less expensive GP test is likely to succeed, thereby avoiding more costly ES.

### Parameter estimation

All relevant parameters are detailed in Table 1 and grouped into the following major categories: (1) cost parameters, including the prices of individual diagnostic tests and expert consultation fees; (2) the turnaround time associated with each test and clinical consultation; (3) test performance metrics, including sensitivity, specificity, and diagnostic yield; (4) AI performance metrics, which inform the derivation of specific transition probabilities in the AI-delegation mode; and (5) parameters used to quantify the expected effectiveness (i.e., QALY). Table 2 provides the estimation for all nontrivial transition probabilities in Figs. 3 & 4 under both expert-alone and AI-delegation modes. As the total probability under each node must sum to one, we present only a representative portion of the distribution. The next few subsections elaborate on how the nontrivial probabilities in Figs. 3 & 4 are estimated.

**Table 1:** Parameters and notations used in framing the PRICE model and their estimations in DD & MCA case study

Parameter Category	**Notation**^1^	Description	Reference Values
Cost	${C_E}$	Expert fee	165 [8], inflation-adjusted 174.28^2^
	${C_0}$	Cost of CMA test	825, inflation-adjusted 871.39 [8]^3^
	$${C_1}$$	Cost of GP test	Ranges from $1450 to $1750 [30]
	${C_2}$	Cost of ES test	4589.4, 45, inflation-adjusted 4847.47 [8]^4^
Turnaround Time	${T_0}$	CMA turnaround time	2 weeks [31, 32]
	${T_1}$	GP turnaround time	4 weeks [33, 34]
	${T_2}$	ES turnaround time	8 weeks [35]
	${T_E}$	Physician consultancy	4 weeks^6^
	${T_{AI}}$	AI decision-making	0 weeks
Test Result	${Y_0}$	Test result of CMA	NA
	${Y_1}$	Test result of gene-panel	NA
	${Y_2}$	Test result of WES	NA
Test Performance	$Y{D_0}$	CMA diagnostic yield	Beta (154, 1382) [8]
	$Y{D_1}$	GP diagnostic yield	Beta (24, 89) [8]
	$YD_2^1$	1^st^ tier ES diagnostic yield	Beta (27, 46) [8]
	$YD_2^2$	2^nd^ tier ES diagnostic yield	0.35 [8]
	$YD_2^3$	3^rd^ tier ES diagnostic yield	Beta (228, 464) [8]
	$S{e_0}$	CMA sensitivity^7^	0.9068 [36]
	$S{p_0}$	CMA specificity	0.9440 [32]
	$$S{e_1} $$	GP sensitivity	0.8960 [37–39]^8^
	$S{p_1}$	GP specificity	0.9250 [33, 34]^9^
	$S{e_2}$	ES sensitivity	0.9593 [40, 41] ^10^
	$S{p_2}$	ES specificity	0.9933 [42, 43] ^11^
AI Performance	$r$	Likelihood of a positive result from GP test	NA
	${P^{AI}}$	AIs precision	0.87 [44]
Disease Prevalence	$p$	DD & MCA prevalence	0.1665 [45]
QALY	${U_{TP}}$	Utility of a true positive	[0.8, 0.9]
	${U_{TN}}$	Utility of a true negative	[0.9, 1.0]
	${U_{FP}}$	Utility of a false positive	[0.7, 0.8]
	${U_{FN}}$	Utility of a false negative	[−0.5, 0], considered worse than death

^1^All the notations’ subscripts in Table 1 are irrelevant to the node numbers shown in Figs. 3 & 4. For simplicity, subscripts 0, 1, and 2 denote CMA, GP and ES, respectively.

^2-4^To account for inflation, the cost can be adjusted by the formula ${C_{adjusted}} = {C_{original}}*{{{{I_{current}}} \over {{I_{base}}}}_{}}$ [46], where ${C_{original}}$ is the cost value provided in this table, ${I_{current}} \& {I_{base}}$ are the Medical Care Consumer Price Index (MCCPI) of any present month and the base month (i.e., when the data is published). The MCCPI captures patients’ out-of-pocket costs and third-party reimbursements (e.g., insurance companies) [47] which is relevant to our context. These index values can be retrieved at FRED [utm_source=chatgpt.com]

^6^A rough estimation based on the expert’s opinion, can vary by different factors (e.g., patients’ urgency level)

^7^Sensitivity (specificity) refers to a test’s ability to correctly identify individuals who truly (does not) have the disease and is quantified as the proportion of true positives (negatives) among all positive (negative) cases [48]. A test may exhibit high sensitivity yet still produce a low diagnostic yield if the disease prevalence in the tested population is low

^8-11^These reference values are the average values taken from multiple sources

**Table 2:** Estimation of ${P_{ij}}$ under various modes

Edge	Mode	Formulation	Explanation
${P_{2,3}}$	Expert-alone & AI-delegation	$1\, or\, 0$	Assume each institution follows a standard protocol.
$P{ _{3,5}}$	Expert-alone & AI-delegation	$P\left( {{Y_0} = 1} \right) = Y{D_0}$	Diagnostic yield of CMA.
${P_{4,7}}$	Expert-alone & AI-delegation	$P\left( {{Y_2} = 1} \right) = YD_2^1$	Diagnostic yield of a first-tier WES.
${P_{10,13}}$	Expert-alone & AI-delegation	$1\, or\, 0$ for expert-alone, 1 for AI-delegation	Assume each institution follows a standard protocol.
${P_{13,15}}$	Expert-alone & AI-delegation	$P\left( { {Y_0} = 0} \right) \cong YD_2^2$	The yield of WES given a negative result from the previous CMA test. Can be directly approximated with the marginal yield of a second-tier WES.
${P_{14,17}}$	Expert-alone	$P({Y_1} = 1 | {Y_0} = 0) \cong Y{D_1}$	The yield of gene-panel given a negative result from the previous CMA test. Can be directly approximated with the marginal yield of gene-panel.
${P_{22,23}}$	Expert-alone	$1\, or\, 0$	Assume each institution follows a standard protocol.
${P_{24,25}}$	Expert-alone	$P\left( { {Y_2} = 1 | {Y_1} = 0,{Y_0} = 0} \right) \cong YD_2^3$	The yield of WES given a negative result from both CMA and gene-panel in the previous tiers of the test. Can be directly approximated with the marginal yield of a third-tier WES.
${P_{1{^{\prime}},2{^{\prime}} }}$	AI-delegation	$P\left( {r > {r^*}} \right) = 1 or 0$	${r^*}$ is always a fixed value for a given patient, thus $P\left( {r > {r^*}} \right)$ is binary (either 1 or 0).
${P_{4{^{\prime}}, 5{^{\prime}}}}$	AI-delegation	$P\left( {{Y_1} = 1\left| { r} \right\rangle {r^*}} \right) = {P^{AI}}$	$P\left( {{Y_1} = 1\left| r \right\rangle {r^*}} \right) = {{P\left( {r > {r^*}, {Y_1} = 1} \right)} \over {P(r > {r^*})}} = {{P\left( {TP} \right)} \over {P\left( {TP + FP} \right)}} = {P^{AI}}$
${P_{8{^{\prime}},9{^{\prime}}}}$	AI-delegation	$P\left( { r > {r^*}, {Y_1} = 0,{Y_0} = 0} \right) \cong YD_2^3$	Since ES yield is irrelevant to AIs prediction, this can still be estimated with the marginal yield of a third-tier WES.

#### Expert-alone scenarios

In real-world clinical settings that rely solely on human expertise, a standardized and institution-wide diagnostic protocol is often applied uniformly across all patients. Under such predefined protocols, the transition probability from a decision node to an action node is deterministic, taking values of either 0 or 1. For example, at the *Decision 1* node in Fig. 3, if the institutional guideline mandates the CMA as the first-tier test, the probability of selecting the CMA is ${P_{2,3}} = 1$. All action nodes represent diagnostic tests that yield either a positive or negative result; the probability associated with a positive result is therefore equivalent to the diagnostic yield of the respective test, which can be estimated as the proportion of positive outcomes relative to the total number of tests administered (e.g., ${P_{3, 5}} = P\left( {{Y_0} = 1} \right) = Y{D_0}$).

Personalized diagnosis would tailor these transition probabilities based on individual patient characteristics, such as phenotypic features, demographics, or clinical presentation, rather than adhering to a fixed diagnostic sequence. In such settings, ${P_{ij}}$ becomes a conditional probability that can be estimated from patient-specific factors and historical clinical data. Estimation may be performed through simple empirical ratios (e.g., the proportion of patients with DD and MCA phenotypes who undergo CMA as the first-tier test) or through more sophisticated predictive models based on patient-level covariates, which can be better achieved through the integration of AI-based models introduced in the next section.

However, owing to the multilayered structure of diagnostic trees, accurately estimating conditional probabilities at deeperlevels becomes increasingly impractical. For example, deriving a probability such as ${P_{24,25}} = P(Y{D_2} = 1 \ | \ patient, Y{D_0} = 0, Y{D_1} = 0)$ may require extensive data that are rarely available in clinical practice. To address this limitation, conditional probabilities at deeper layers can be approximated via marginal probabilities as a practical solution. When constructing personalized diagnostic pathways, it is thus essential to balance the trade-off between predictive accuracy and computational feasibility when deciding whether to adopt marginal or conditional probability estimation.

#### AI-delegation branch

The AI-delegation branch introduces two distinct probability types not presented in the expert-alone mode: the probability associated with the AI’s result node (${P_{1{^\prime}, 2{^\prime}}}$) and the GP’s diagnostic yield conditioned on the AI’s prediction (${P_{14{^\prime}, 17{^\prime}}}$). When the prediction score, *r*, varies at the population level, the corresponding probability ${P_{1{^\prime}, 2{^\prime}}} = P\left( {r \ge {r^*}} \right)$ lies within the range of (0,1). However, when the prediction is conditioned on a specific patient, i.e., ${P_{1{^\prime}, 2{^\prime}}} = P(r \ge {r^*} \ | \ patient)$, the prediction result *r* becomes a fixed value on the basis of the patient’s input data and the AI model; thus, ${P_{1{^\prime}, 2{^\prime}}} = 0, or \ 1$ depending on the threshold value ${r^*}$. Furthermore, the diagnostic yield of the GP $({P_{14{^\prime}, 17{^\prime}}})$ under the AI-delegated branch $r \ge {r^*}$ differs from that in the expert-alone mode. Rather, it reflects AI performance and can be formally defined as $P ( {{y_1} = 1 | { r} > {r^*}} ) = {{P\left( {True\, Positive} \right)} \over {P\left( {Predicted\, Positive} \right)}}$ (i.e., AI precision). Assuming that the AI model is unbiased, its predictive performance should be treated as independent of specific patient phenotypes or demographics. The subsequent nodes under the $r < {r^*}$ branch revert to expert judgment and remain unaffected by the AIs performance; thus, the transition probabilities follow the same estimation as in the expert-alone mode.

### Expected QALY

Recall the *outcome state* defined in section “Cost effectiveness analysis”. In this case study, all diagnostic tests considered produce dichotomous outcomes, leading to either positive or negative diagnostic results as the final outcome (i.e., ${O_i} = 1 \,or \,0$). In this example, the expected utility of the outcome state ${S_i}$ should consider the expected utility of both cases of a true diagnosis (i.e., true positive $\left( {{U_{TP}}} \right)$ or true negative $\left( {{U_{TN}}} \right)$) and a false diagnosis (i.e., false positive $\left( {{U_{FP}}} \right)$ or false negative $({U_{FN}})$), which can be mathematically defined as

$$ E\left[ {U\left( {{S_i}} \right)} \right] = PP{V_i}_*{U_{TP}} + \left( {1 - PP{V_i}} \right)*{U_{FP}} \ for\ {\mkern 1mu} {O_i} = 1 $$

$$ E\left[ {U\left( {{S_i}} \right)} \right] = NP{V_i}*{U_{TN}} + \left( {1 - NP{V_i}} \right)*{U_{FN}} \ for \ {\mkern 1mu} {O_i} = 0 $$

Existing literature suggests several general principles for assigning utility weights to diagnostic outcomes which we may apply directly. A true positive or negative result is often viewed positively, often set to a value at or near perfect utility $\left( {e,g. {U_{TP}} = 1} \right)$ [28, 49]. False negatives, on the contrary, are often associated with significantly negative utility values due to serious consequences such as delayed or missed treatment $({U_{FN}} < 0)$ [50–52]. True positive and false positive outcomes both carry positive utility weights $({U_{TP}} > 0, {U_{FP}} > 0)$ with ${U_{TP}} > {U_{FP}}$ [53, 54] The positive predictive value (PPV) and negative predictive value (NPV) reflect the accuracy of the final diagnostic results, which can be derived from the disease prevalence (*p*), the sensitivity (*Se*) and specificity (*Sp*) of the diagnostic tests to yield the final results (see Table 1) [55]:

$$PPV = {{Se*p} \over {Se*p + \left( {1 - Sp} \right)*\left( {1 - p} \right)}};$$

$$NPV = {{Sp*\left( {1 - p} \right)} \over {Sp*\left( {1 - p} \right) + \left( {1 - Se} \right)*p}}.$$

Since each diagnostic strategy outlined in section “Tree representation” leads to multiple possible outcomes, the expected QALY is calculated as the probability-weighted sum of the utilities across all outcome states.

### Cost-effectiveness analysis under various scenarios

In this section, we conduct a probabilistic sensitivity analysis via Monte Carlo simulation to evaluate how parameter uncertainty influences cost-effectiveness outcomes. For each simulation run, a unique set of parameter values is sampled from appropriate probability distributions to reflect their natural variability, while certain parameters with minimal uncertainty are held constant. Table 3 lists all parameters together with their distributions or fixed estimates used in the simulation. With a total of 1000 simulations executed, we generate a dataset that records the sampled parameter values and the corresponding cost-effective results (i.e., cost per QALY for the next 1 year following the start of diagnostic process). Since the cost of genetic tests can differ significantly across labs, healthcare systems and even among patients themselves depending on condition complexity or insurance coverage, test-cost parameters are treated as the main source of variation. Variability in AI performance is also explicitly considered to capture the full spectrum of cost-effective outcomes across different performance levels. Table 4 summarized the basic statistics of cost per QALY for each scenario. In the following two subsections, we provide examples within real-world clinical contexts to visualize the simulation results and demonstrate how the findings can be interpreted to inform diagnostic decision-making.

**Table 3:** Parameters used in Monte Carlo Simulation

Notation	Description	Value/Distribution
${C_E}$	Expert fee	174.28
${C_0}$	Cost of CMA test	Lognormal (6.8628, 0.3)
${C_1}$	Cost of GP test	Lognormal (3.1241, 0.4)
${C_2}$	Cost of ES test	Gamma (4, 1211.8675)
${T_0}$	CMA turnaround time	Random sample between 1 to 3 weeks
${T_1}$	GP turnaround time	Random sample between 2 to 6 weeks
${T_2}$	ES turnaround time	Random sample between 4 to 12 weeks
${T_E}$	Physician consultancy	4 weeks
${T_{AI}}$	AI decision-making	0 weeks
$Y{D_0}$	CMA diagnostic yield	Beta (154, 1382)
$Y{D_1}$	GP diagnostic yield	Beta (234, 1871)
$YD_2^1$	1^st^ tier ES diagnostic yield	Beta (27, 46)
$YD_2^2$	2^nd^ tier ES diagnostic yield	Beta (35, 65)
$YD_2^3$	3^rd^ tier ES diagnostic yield	Beta (228, 464)
$S{e_0}$	CMA sensitivity^7^	0.9068
$S{p_0}$	CMA specificity	0.9440
$$S{e_1} $$	GP sensitivity	0.8960
$S{p_1}$	GP specificity	0.9250
$S{e_2}$	ES sensitivity	0.9593
$S{p_2}$	ES specificity	0.9933
${P^{AI}}$	AIs precision	Uniform (0.1, 0.9)
*p*	DD & MCA prevalence	0.1665
${U_{TP}}$	Utility of a true positive	Uniform (0.8, 0.9)
${U_{TN}}$	Utility of a true negative	Uniform (0.9, 1.0)
${U_{FP}}$	Utility of a false positive	Uniform (0.7, 0.8)
${U_{FN}}$	Utility of a false negative	Uniform (−0.5, 0), considered worse than death

#### Example 1

**Fig. 5:**
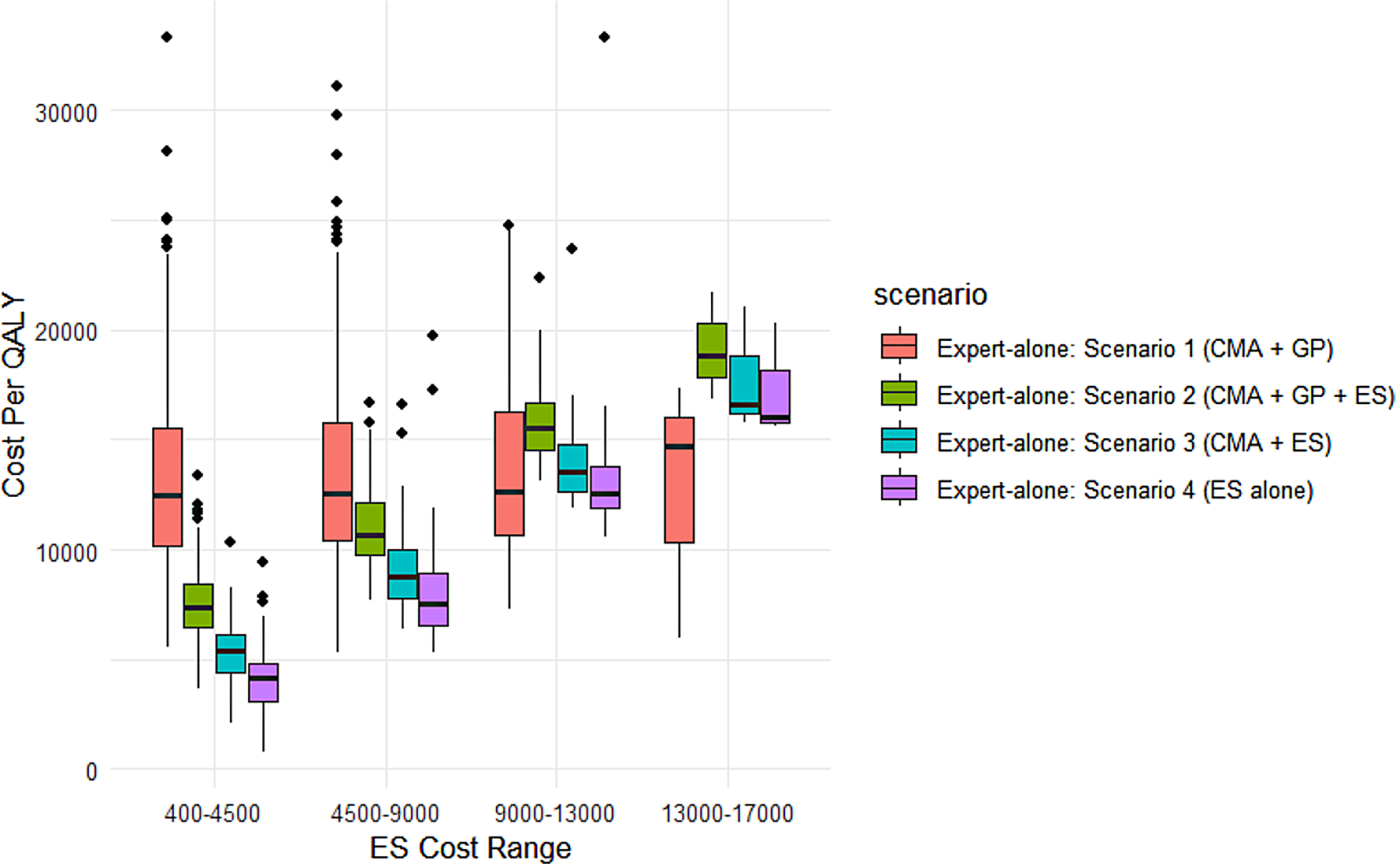
Cost per QALY vs. ES cost range, expert-alone scenarios. Cost Per QALY of expert-alone diagnostic strategies across varying intervals of ES cost. The parameter values are mostly taken from the reference distributions and fixed values in Table 1, except the cost of CMA $\left( {{C_0} \sim lognorm\left( {{\rm{log}}(1000} \right) - 0.045, 0.3} \right))$, gene-panel $\left( {{C_1} \sim lognorm\left( {\log \left( {1600} \right) - 0.08, 0.4} \right)} \right)$, and ES $\left( {{C_2} \sim gamma\left( {4, 1211.87} \right)} \right)$

Consider a hypothetical case of a 2-year-old patient presenting with DD & MCA phenotypes and four health institutions, each implementing one of the expert-alone diagnostic strategies described in section “Tree representation” as their standard clinical protocol. Figure 5 illustrates how the cost per QALY (measured one year after the initiation of diagnostics) varies with the price of ES, the most advanced yet costly genetic test. The *ES-alone* pathway in scenario 4 emerges as the most cost-effective strategy when ES is priced within the lower cost intervals. As ES gets significantly more expensive, the CMA + GP path in scenario 1 becomes marginally more cost-effective than ES-alone strategy. Taken together, these results suggest that while the ES-alone pathway remains the best option in most cases, CMA + GP strategy should be considered when the cost of ES is high enough.

#### Example 2

**Fig. 6:**
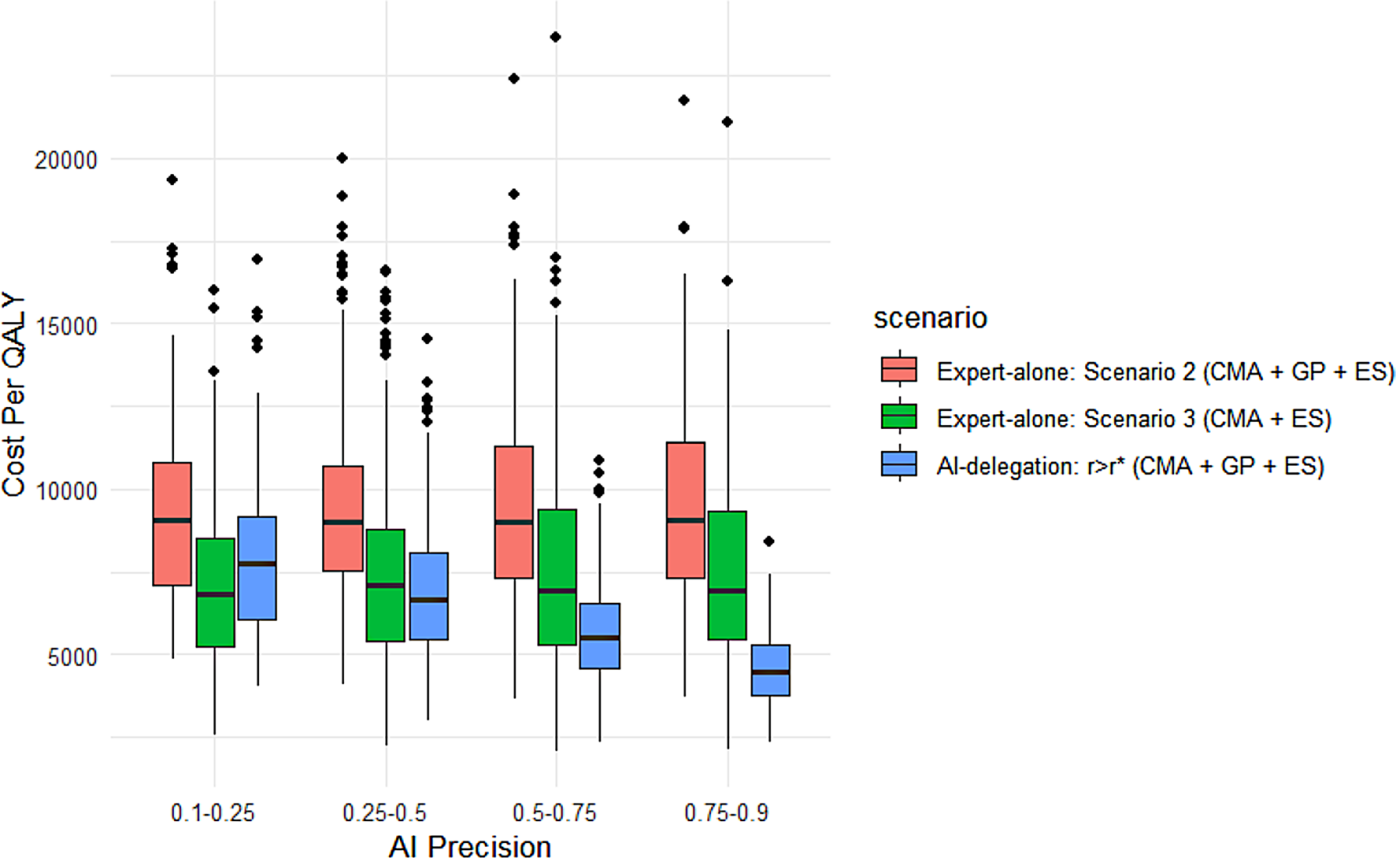
Cost Per QALY vs. AI Precision. Cost per QALY of the AI-delegation mode compared to those of scenarios 2 and 3 under the expert-alone mode across varying values of AI precision with distribution *Unif* (0.1, 0.9)

**Fig. 7:**
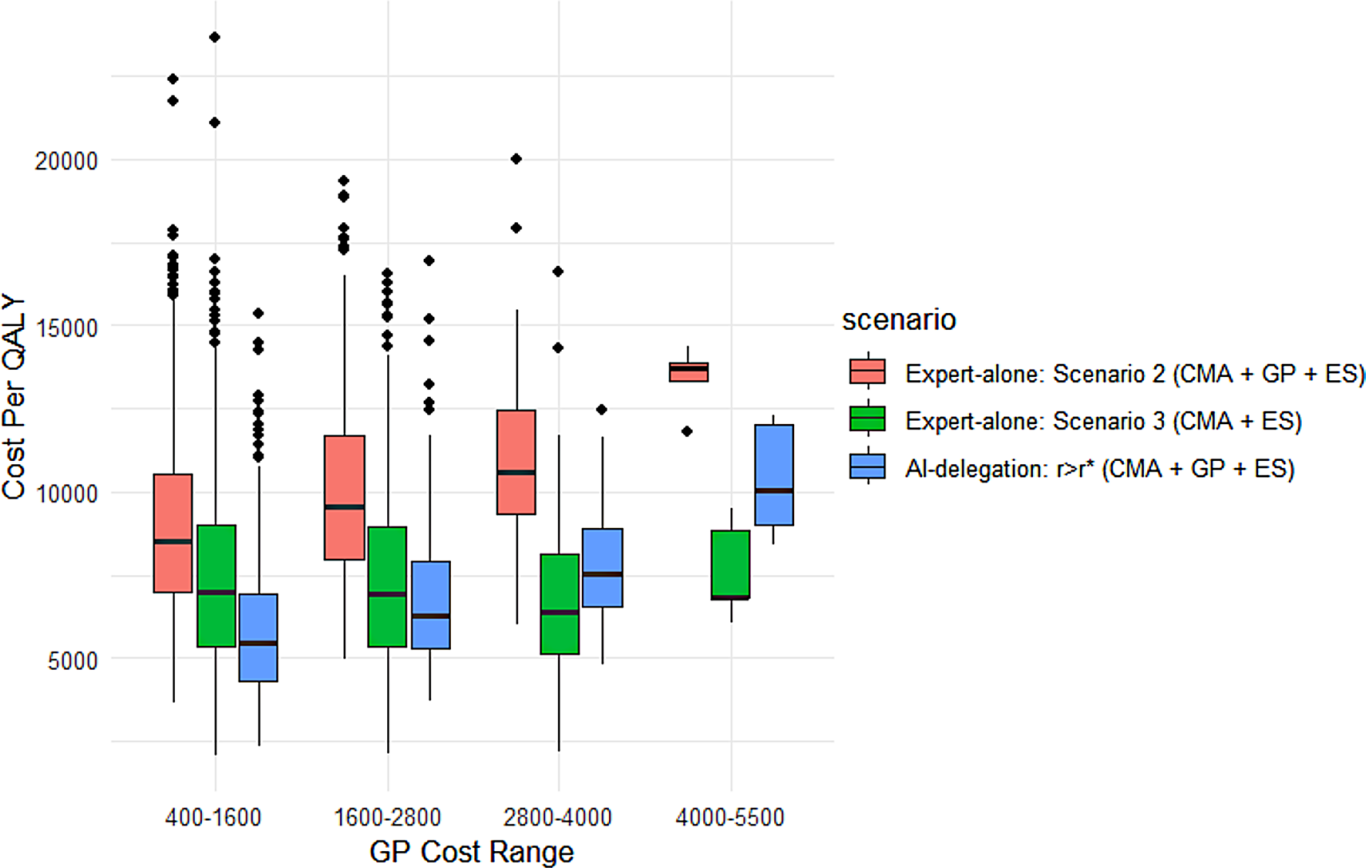
Effective Cost vs. Gene-panel Cost, AI-delegation. Cost per QALY of the AI-delegation mode compared to scenarios 2 and 3 under the expert-alone mode across varying intervals of gene-panel cost

Now consider a hypothetical situation in which another healthcare institution fully employs an AI-assisted diagnostic model (i.e., the AI-delegation mode as shown in Fig. 4), and assume that the patient will be directed to the $r \ge {r^*}$ branch corresponding to a partially automated CMA + GP + ES pathway given his (her) specific profile. As illustrated in Fig. 6, the cost per QALY of the AI-automated pathway declines steadily with higher AI precision increases, eventually outperforming both expert-alone scenarios 2 and 3 once the precision reaches a moderate level (e.g., 0.5 ~ 0.75). These results indicate that a sufficiently accurate AI model can provide a more cost-effective and personalized alternative to conventional expert-based strategies. From a decision-making perspective, this AI-delegated strategy offers an appealing middle ground for caregivers seeking to incorporate ES into the diagnostic process while deferring its use when possible. However, the relative cost of GP should also remain an important determinant: Figure 7 shows that when the cost of GP is high enough, the AI delegation mode would be markedly less cost-effective than the CMA + ES path in expert-alone scenario 3. Accordingly, the AI-delegated approach is most advantageous when GP costs are maintained within a moderate range.

**Table 4:** Cost per QALY - summary statistics

Scenario	Mean	Median	Min	Max	Standard Deviation	95% Interval
Expert-alone Scenario 1 (CMA + GP)	12429.32	11860.56	4180.76	29753.90	3869.23	[12189.50, 12,669.14]
Expert-alone Scenario 2 (CMA + GP + ES)	9332.44	8941.59	3557.93	24637.52	2767.94	[9160.88, 9503.00]
Expert-alone Scenario 3 (CMA + ES)	7254.80	6839.37	1649.04	23659.75	2787.99	[7082.00, 7427.60]
Expert-alone Scenario 4 (ES alone)	6054.06	5655.97	457.03	21715.51	2903.58	[5874.10, 6234.03]
AI-delegation *r* > r* (CMA + GP + ES)	6129.07	5787.34	1976.72	22280.84	2154.06	[5995.56, 6262.58]

## Discussion

In this section, we discuss generalizability of the PRICE analysis framework through another example, re-examine the underlying assumptions and outline future directions for real-world implementations. 

### Structural heart disease diagnosis example

**Fig. 8:**
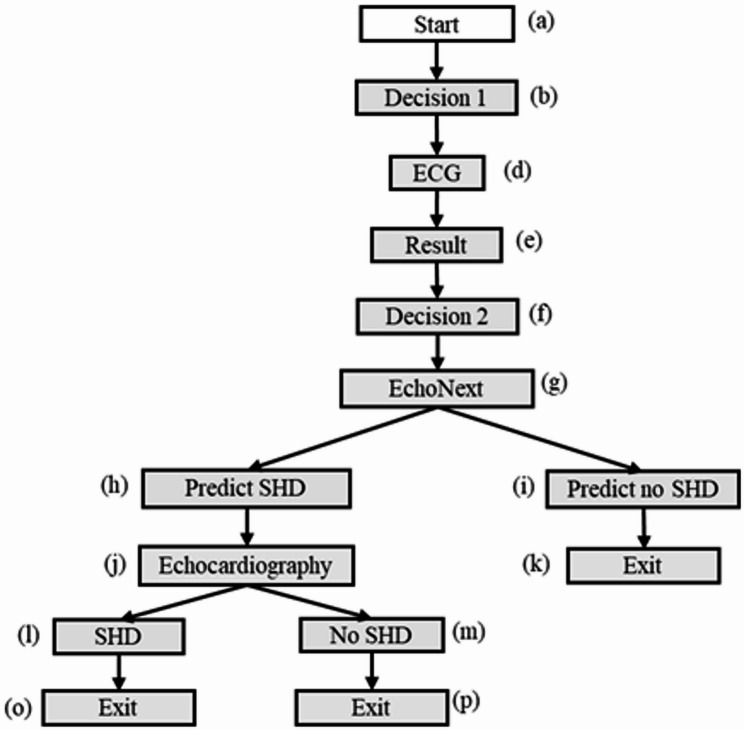
Structural heart disease (SHD) diagnostic process a simplified process for structural heart disease diagnosis. Patients first go through an electrocardiogram (ECG), and the results are analyzed by the ai model *EchoNext*, which classifies the likelihood of structural heart disease (SHD). If shd is predicted, the patient proceeds to echocardiography; otherwise, the diagnostic process concludes

To demonstrate the generalizability of the PRICE framework on other diagnostic processes, we show another example of structural heart disease diagnosis (SHD) [56] in this subsection. In this study, a deep learning model, *EchoNext*, was developed to analyze the standard electrocardiogram (ECG) data to determine whether patients should go through echocardiography (e.g. an expensive procedure) for confirmation of SHD. Figure 8 illustrates the tree structure of the diagnostic process of SHD, involving *EchoNext* as AI node, and Table 3 summarizes all the relevant parameters that will be used in the framework. The integration of AI in this procedure plays a take-over role rather than the delegation role as in *DD & MCA* case. In this diagnostic tree, the only non-trivial transition probabilityies in this tree is are ${P_{g,h}} = P(predicted \ SHD \ | \ ECG) \approx 0.27$, the probability that *EchoNext* predicts SHD for an average patient (this probability should nonetheless be either 0 or 1 given a specific patient’s ECG, following the same rationale as the probability $P\left( {r > {r^*}} \right)$ in the AI-delegation mode); and ${P_{j,l}} = P(SHD \ exists \ | \ predicted \ SHD)$, the probability that the patient actually has SHD given the prediction by *EchoNext*. Similar to the probability ${P_{4{^\prime}, 5{^\prime}}}$ of AI-delegation mode in Table 2, ${P_{j,l}}$ can be further derived as

$$\eqalign{& {P_{j,l}} \cr & \,\,\, = P(True{\mkern 1mu} \ Positive \ | \ Predicted \ {\mkern 1mu} Positive) \cr & \,\,\, = {{P\left( {TP} \right)} \over {P\left( {TP + FP} \right)}} = {P^{next}} = 0.74, \cr} $$

which is the precision of *EchoNext*.

With all the parameters defined in Table 5, the *BECC* algorithm for expected cost can be directly applied to this diagnostic process. The measurement of QALY, however, requires some extra attention. Recall the general formula for QALY in section “Cost effectiveness analysis”, the outcome states in Figure 8 would be at node (k), which ends with a “*Predict no SHD”* result from the AI; and node (o) and (p) which capture the results from echocardiography. Similar to a diagnostic test, the expected utility assigned to node (k) should account for both true negative and false negative classifications generated by the AI. Since AI’s decision is embedded within the diagnostic pathway, its impact extends directly to downstream health outcomes (e.g., delayed or missed treatment following a *“Predict no SHD”* result). Consequently, the assignment of utility weights at this node may follow the same principles applied to diagnostic test outcomes, as summarized in section “Expected QALY”. In contrast, echocardiography is widely regarded as a *gold standard*; therefore, it is typically assumed to yield neither false positive nor false negative. A single utility weight (${U_{TP}} \,or \,{U_{TN}}$) can thus be directly applied to nodes (o) and (p).

**Table 5:** Parameters - structural heart disease

Parameter Category	Notation	Description	Reference Value
Cost	$${C_E}$$	Expert fee	165 [8], inflation-adjusted 174.28
	$${C_{ECG}}$$	Cost of ECG	National average price $708 [57]
	$${C_{echo}}$$	Cost of echocardiography	Nation average price $1852 [58]
	$${C_{next}}$$	Cost of EchoNext	Assume patients do not pay any cost for AI predictions
Turnaround Time	$${T_{ECG}}$$	Turnaround time of ECG	ECG results are usually obtained immediately once scanning is finished
	$${T_{echo}}$$	Turnaround time of echocardiography	2 weeks for scheduling; results are typically available within 24 hours [59]
	$${T_{next}}$$	Turnaround time of EchoNext	Immediately
	$${T_E}$$	Turnaround time of cardiologist consultancy	Median time around 4 weeks [59]
AI Performance	$${P_{next}}$$	EchoNext precision	0.74 [56]
Test Performances	$$Y{D_{echo}}$$	Echocardiography diagnostic yield	0.29 for general outpatient [60]
	$$S{e_{echo}}$$	Echocardiography sensitivity	0.726 [53]
	$$S{p_{echo}}$$	Echocardiography specificity	0.807 [53]
Disease Prevalence	$${p_{SHD}}$$	The prevalence of SHD	0.41 [53]
QALY Related	$${U_{TP}}$$	Utility of a true positive	[0.8, 0.9]
	$${U_{TN}}$$	Utility of a true negative	[0.9, 1.0]
	$${U_{FP}}$$	Utility of a false positive	[0.7, 0.8]
	$${U_{FN}}$$	Utility of a false negative	[−0.5, 0]
Transition Probabilities	$${P_{g,h}}$$	The probability that EchoNext predicts SHD based on ECG results	0.27
	$${P_{j,l}}$$	The probability that SHD actually exists, given that EchoNext predicts SHD. This is basically the precision of EchoNext	0.74

### Challenges, assumptions, and ethical considerations

As AIs involvement plays a primary role in achieving *personalized* cost estimation, our framework relies on the presumption that AI-assisted decision-making tools have been fully implemented in clinical workflows with pre-defined AI prediction threshold r*. Because the cost-effective results can be sensitive to the chosen threshold values, selecting appropriate thresholds for real-world implementation should extend beyond traditional AI performance-based approaches (e.g., decision curve analysis [61]) and incorporate cost-effectiveness perspectives. In our proof-of-concept, we assume that decisions are triggered solely by AI prediction results; while in reality, clinicians are likely to be involved to prevent misdiagnoses that could lead to serious consequences. Although a more realistic scenario can also be modeled within the PRICE framework, the AI-delegation mode in our case study only focuses on triggering automated test ordering for simplicity.

Another major assumption underlying PRICE AI-delegation mode is the unbiasedness of AI models, meaning their performance would be consistent across all patients and subgroups (e.g., ethnic minorities or low-income populations). It is important to clarify that this assumption does not imply uniform predictive outcomes across patients. As AI models generate individual predictions, each patient receives a unique prediction result based on their specific data inputs. In this context, *unbiasedness* refers to the consistency of overall model performance metrics (e.g., accuracy) across all patient subgroups. However, this is rarely achieved in current AI development, since many AI systems are trained on datasets that underrepresent certain demographic groups such as ethnic minorities or low-income population [62]. These disparities can lead to systematic errors and, in turn, bias the cost-effectiveness analysis for underrepresented groups. Although this paper is not going to address this issue involved in AI development, we believe stratified analyses should be conducted to capture parameter heterogeneity across demographic groups, such that potential inequities can be identified and avoid exacerbating healthcare disparities before AI tools being deployed clinically. In cases where training data biases are substantial, the AI-assisted PRICE analysis should only be limited to subgroups where reliable performance can be demonstrated.

From a computational standpoint, the recursive structure of PRICE maintains a time complexity *O(N)* that is linear to the number of nodes in the tree (recall Section “Algorithm for Expected Cost Computing”). This linear complexity enables scalability to complex diagnostic workflows without an exponential growth in processing time, maintaining operational feasibility for practical implementation. However, another critical challenge in deploying the PRICE framework in real-world clinical settings lies in obtaining reliable and comprehensive data to inform personalized model parameters, such as conditional transition probabilities and individual cost estimates. Estimating transition probabilities between nodes, such as test selection probabilities or diagnostic yields, requires access to detailed, longitudinal patient records, which are often incomplete or inconsistently documented [42, 43]. Patient-level covariates (e.g., demographic factors, clinical history, etc.) needed for personalized probability estimation may also be unavailable or non-standardized across healthcare systems [63, 64]. In practice, marginal probabilities might be needed to approximate the conditional probabilities in deeper layers. Parameters like costs and turnaround times also vary widely across institutions, regions, and payer systems, raising additional difficulties and equity concerns [65]. For example, rural clinics or lower-income hospitals may face longer wait times, limited test availability, or disproportionately high costs due to logistical barriers [66, 67]. Cost transparency adds another layer of complexity: healthcare expenses are often opaque due to insurer involvement, who may prioritize minimizing expenditures over ensuring clinical effectiveness.

In summary, the successful application of the PRICE framework in real-world clinical settings requires several fundamental conditions. First, the relevant input data, such as cost parameters, and diagnostic yields needed to estimate transition probabilities, must be credibly obtained or reliably approximated. When AI tools are incorporated into the diagnostic process, it is critical to ensure that their predictive performance is unbiased for the target patient population, with predefined threshold values established before deployment. As any biases in AI predictions could distort the estimated cost-effectiveness results computed by the PRICE framework, relying on conventional expert-driven decision making may provide a more equitable basis if AI’s unbiasedness cannot be satisfied.

### Limitation and future directions

While the PRICE framework provides a theoretical foundation, future improvements should emphasize empirical validation and clinical translation for real-world applicability. A subsequent roadmap can be envisioned as follows:

1. Explicitly account for insurance policies and distinguish payers’ reimbursements from patients’ out-of-pocket expenses, given the variability across insurance policies and patient groups.
2. Stakeholder interviews with clinicians, patient representatives and health economists to refine the framework’s usability and interpretability.
3. Pilot implementations within a specific diagnostic workflow and patient cohort to assess the framework’s real-world feasibility; parameterizing with patient-level data (e.g., turnaround times, out-of-pocket costs etc.) to validate whether anticipated cost-effectiveness aligns with observed outcomes. Appropriate imputation methods [68, 69] may be required to address missing or incomplete medical data. To ensure data security and privacy, the framework could be deployed locally on patient-controlled or institution-managed devices, allowing computation to occur without transferring identifiable patient data externally.
4. To better visualize the conceptual framework, we have also developed a web-based prototype (http://165.22.13.117:4833/) that enables users to construct customized diagnostic trees. This interactive platform supports real-time cost estimation via the *BECC* algorithm and allows dynamic adjustments of nodes and parameters. At present, the tool serves only as a proof of concept; future real-clinics implementation will require pilot testing, expert feedback, and exploration of integration into shared decision-making processes.

Another limitation of the present framework is its focus on the diagnostic decision process without modeling subsequent health states such as treatment response, disease progression, or long-term outcomes. In principle, the decision-tree structure of the PRICE framework could be extended with a Markov modeling structure where post-diagnostic states are defined to capture lifetime costs and effectiveness, thereby linking diagnostic accuracy with downstream clinical and economic consequences [70, 71]. However, such integration would require longitudinal parameters over a long-term horizon, which are typically unavailable at the diagnostic stage.

## Conclusion

We introduced PRICE, a *Personalized Recursive Intelligent Cost-Effectiveness* framework designed to model and evaluate diagnostic pathways. The framework demonstrates, through simulation and conceptual analysis, how recursive decision structures can enable more flexible and individualized cost-effectiveness estimation compared to traditional static models. The case studies presented illustrate the framework’s adaptability across various clinical contexts, highlighting its potential to evaluate AI-enabled procedures alongside conventional testing strategies. It is however important to emphasize that the current findings are conceptual rather than empirical, relying on simulated parameter values rather than real-world patient data. The current implementation of this framework should therefore be interpreted as illustrative and conceptual, with more empirical validations needed for real-world applicability.

We also highlighted several key assumptions and challenges underlying the current proof-of-concept. PRICE assumes that AI-assisted decision tools are unbiased and perform consistently across patient subgroups, an idealized condition seldom achieved in real-world AI development due to disparities in training data. The real-world implementation of the framework also depends on the availability of standard and transparent parameter values (e.g., genetic test costs, diagnostic yields, turnaround times etc.) which may vary substantially across institutions and populations. Future development of this framework should focus on relaxing the assumptions through subgroup-specific calibration, improving data integration, and validating the model empirically via pilot studies, stakeholder co-design, and real-world clinical trials.

## Data Availability

The source codes of the Monte Carlo simulation and the web-based tool developed for this study are available at [(https://github.com/adelina120/DD-MCA-Diagnosis-Process-Cost-Model/blob/main/Monte_Carlo.Rmd)] and [https://github.com/stormliucong/PRICE-React-D3-Tree]. The web-based tool for constructing diagnostic trees is also published at http://165.22.13.117:4833/. No additional research data involved.
